# Genome-wide characterization of the seasonal H3N2 virus in Shanghai reveals natural temperature-sensitive strains conferred by the I668V mutation in the PA subunit

**DOI:** 10.1038/s41426-018-0172-4

**Published:** 2018-10-23

**Authors:** Dong Wei, De-Ming Yu, Ming-jie Wang, Dong-hua Zhang, Qi-jian Cheng, Jie-Ming Qu, Xin-xin Zhang

**Affiliations:** 1 Research Laboratory of Clinical Virology, Ruijin Hospital, Shanghai Jiaotong University School of Medicine, Shanghai, China; 2 Department of Infectious Diseases, Institute of Infectious and Respiratory Diseases, Ruijin Hospital, Shanghai Jiaotong University School of Medicine, Shanghai, China; 3 Department of Respiratory Diseases, Ruijin Hospital North, Shanghai Jiaotong University School of Medicine, Shanghai, China; 4 Department of Respiratory Diseases, Ruijin Hospital, Shanghai Jiaotong University School of Medicine, Shanghai, China; 5 Clinical Research Center, Ruijin Hospital North, Shanghai Jiaotong University School of Medicine, Shanghai, China

## Abstract

Seasonal H3N2 influenza viruses are recognized as major epidemic viruses, exhibiting complex seasonal patterns in regions with temperate climates. To investigate the influence of viral evolution and mutations on the seasonality of influenza, we performed a genome-wide analysis of samples collected from 62 influenza A/H3N2-infected patients in Shanghai during 2016–2017. Phylogenetic analysis of all eight segments of the influenza A virus revealed that there were two epidemic influenza virus strains circulating in the 2016–2017 winter season (2016–2017win) and 2017 summer season (2017sum). Replication of the two epidemic viral strains at different temperatures (33, 35, 37, and 39 °C) was measured, and the correlation of the mutations in the two epidemic viral strains with temperature sensitivity and viral replication was analyzed. Analysis of the replication kinetics showed that replication of the 2016–2017win strains was significantly restricted at 39 °C compared with that of the 2017sum strains. A polymerase activity assay and mutational analysis demonstrated that the PA I668V mutation of the 2016–2017win viruses suppressed polymerase activity in vitro at high temperatures. Taken together, these data suggest that the I668V mutation in the PA subunit of the 2016–2017win strains may confer temperature sensitivity and attenuate viral replication and polymerase activity; meanwhile, the 2017sum strains maintained virulence at high temperatures. These findings highlight the importance of certain mutations in viral adaptation and persistence in subsequent seasons.

## Introduction

Seasonal influenza is responsible for an average of approximately 1 billion infections worldwide, including 250,000 to 500,000 deaths annually. Increasing evidence has suggested that seasonal influenza patterns are highly diverse in different zones, especially in Asia, where these patterns can exhibit semiannual or annual epidemic cycles. The complexity of this pattern has hindered the establishment of effective routine immunization programs^1^. China can be divided into the following three regions according to the seasonality of influenza viruses: the northern provinces experience epidemics during winter, the southern provinces experience epidemics that peak during spring, and provinces at intermediate latitudes experience semi-annual epidemics. Shanghai belongs to the category of provinces at intermediate latitudes, and influenza A epidemics, particularly seasonal A/H3N2 and A/H1N1 viruses, usually appear between autumn and spring, with the influenza activity peaking after October^2,3^.

Between 2014 and 2016, only sporadic influenza infections were reported during summer in Shanghai. However, in 2017, the number of infections increased substantially between May and September among inpatients at Ruijin Hospital. To further examine the influenza epidemics during the summer of 2017 in Shanghai, we compared virus samples obtained during the 2016–2017 winter season in Shanghai with virus samples obtained during the summer season. An etiological and viral replication analysis was performed to investigate the effects of the mutations in the internal gene segments of the influenza A virus during 2017. The influenza A virus contains eight gene segments, namely, basic polymerase 2 (PB2), basic polymerase 1 (PB1), acidic polymerase (PA), hemagglutinin (HA), nucleoprotein (NP), neuraminidase (NA), matrix (M), and nonstructural protein (NS). Antigenic drift in the surface glycoproteins (e.g., HA and NA) can facilitate viral evasion of the host’s immune system^4^. Certain mutations in the polymerases may affect temperature sensitivity, which is characterized by restricted (≥100-fold) replication at a temperature of 39 °C or higher^5–8^.

The aim of this study was to compare the complete genome sequences of two sequential influenza epidemic viruses and to investigate whether the genetic differences in the two epidemic viruses were correlated with temperature sensitivity and viral replication. The results presented here suggest that the mutation in PA I668V may confer temperature sensitivity and attenuate the polymerase activity of the influenza virus, changing the seasonality and adaptation of the virus.

## Results

### Clinical characterization of patients infected with the H3N2 influenza virus during 2016–2017

Sixty-two patients were diagnosed with a H3N2 influenza virus infection from Nov. 2016 to Sep. 2017: 28 patients in the 2016–2017 winter season (from Nov. 2016 to Mar. 2017) and 34 patients in the 2017 summer season (from May 2017 to Sep. 2017). We divided all of the viral strains isolated from influenza-infected patients into two groups: the 2016–2017win virus strains and 2017sum virus strains. The median age of the hosts of the 2016–2017win strains was 66, with ages ranging from 19 to 94, while the median age of the hosts of the 2017sum strains was 70, with ages ranging from 33 to 90 years. Of the 62 patients, 33 (53.2%) were male and 48 (77.4%) were elderly (≥60 years). Influenza-like symptoms (i.e., temperature ≥ 38 °C, cough, or sore throat) were observed in all patients who were subsequently diagnosed with influenza A by real-time RT-PCR. No significant differences were observed in gender, age, underlying condition, or outcome between the two groups. The vaccination rate in Shanghai during 2016–2017 is unclear, but previous studies have suggested that the rate might be relatively low (approximately 2–16%)^3,9,10^. The clinical characteristics of these patients were excluded as possible causes of the two different epidemics. The detailed patient characteristics are described in Table 1.

**Table 1:** Demographic characteristics and clinical features of 62 inpatients with influenza infection from 2016–2017

Characteristics	Inpatients during Nov. 2016– Mar. 2017	Inpatients during May. 2017– Sep. 2017	*P* value
Age (years), median age (range)	66 (19–94)	70 (33–90)	0.390^a^
Males/females	16/12	23/11	0.438^b^
Elder(≥60), %	71.4	82.4	
Fever, %	92.9	88.2	
Chest-X ray abnormalities, %	85.7	82.4	
Underlying condition, %	75.0	76.5	0.893^b^
Outcome (cured/dead, %)	82.1/7.1	82.4/8.8	0.809^b^
Co-infection with bacteria or fungus, %	28.6	20.6	

^a^*P* value was calculated with one-way analysis of variance

^b^*P* values were calculated with Chi-square test

### Genome-wide phylogenetic analysis indicated that the 2017sum virus strains clearly differed from the 2016–2017win virus strains, with multiple mutations in several segments

**Fig. 1:**
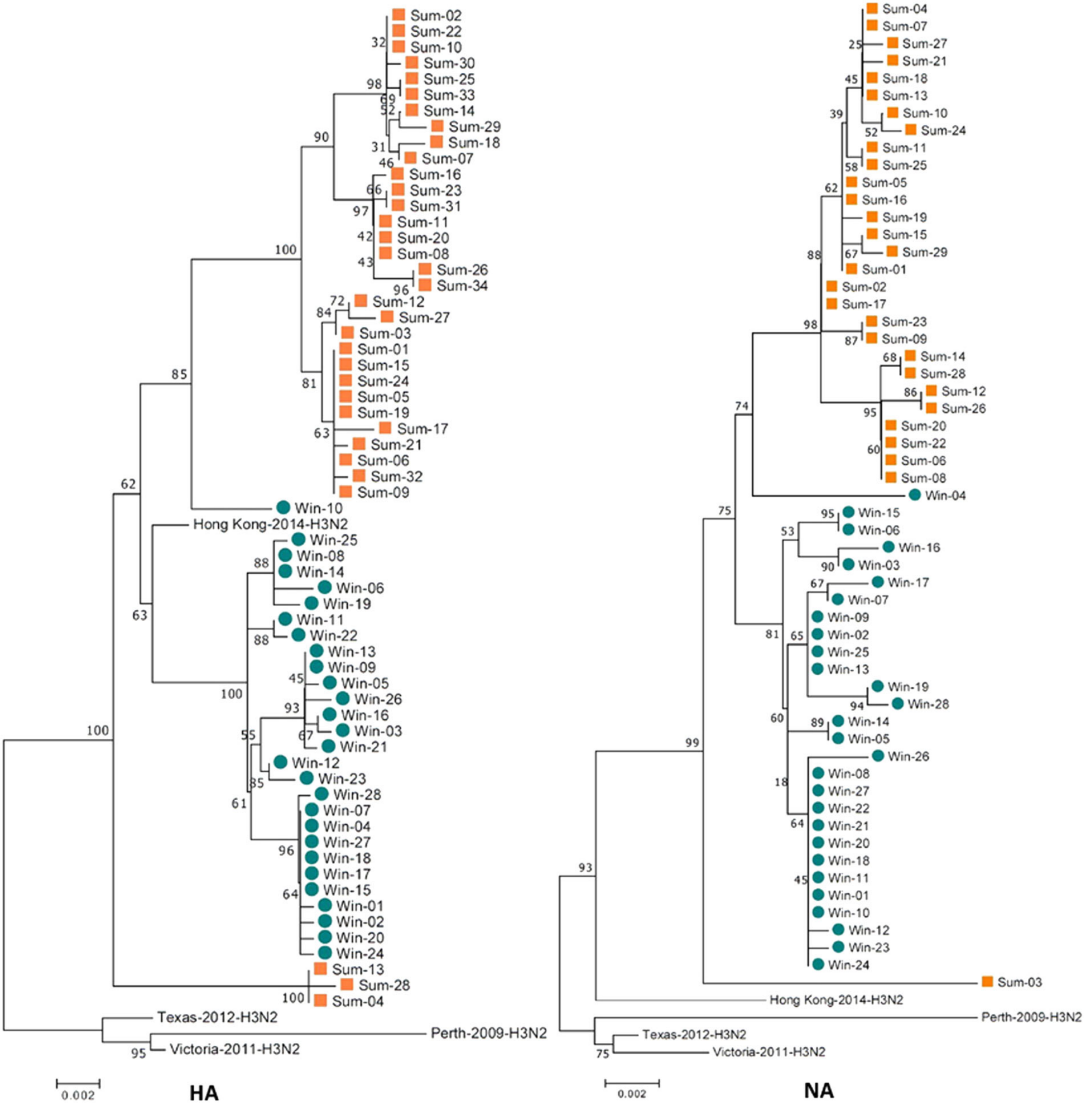
Phylogenetic analysis of the HA and NA segments circulating between Nov. 2016 and Sep. 2017. The 2016–2017win virus strains are designated Win01–28, and the 2017sum virus strains are designated Sum01-34. The 2017sum virus strains significantly differed from the 2016–2017win virus strains

**Fig. 2:**
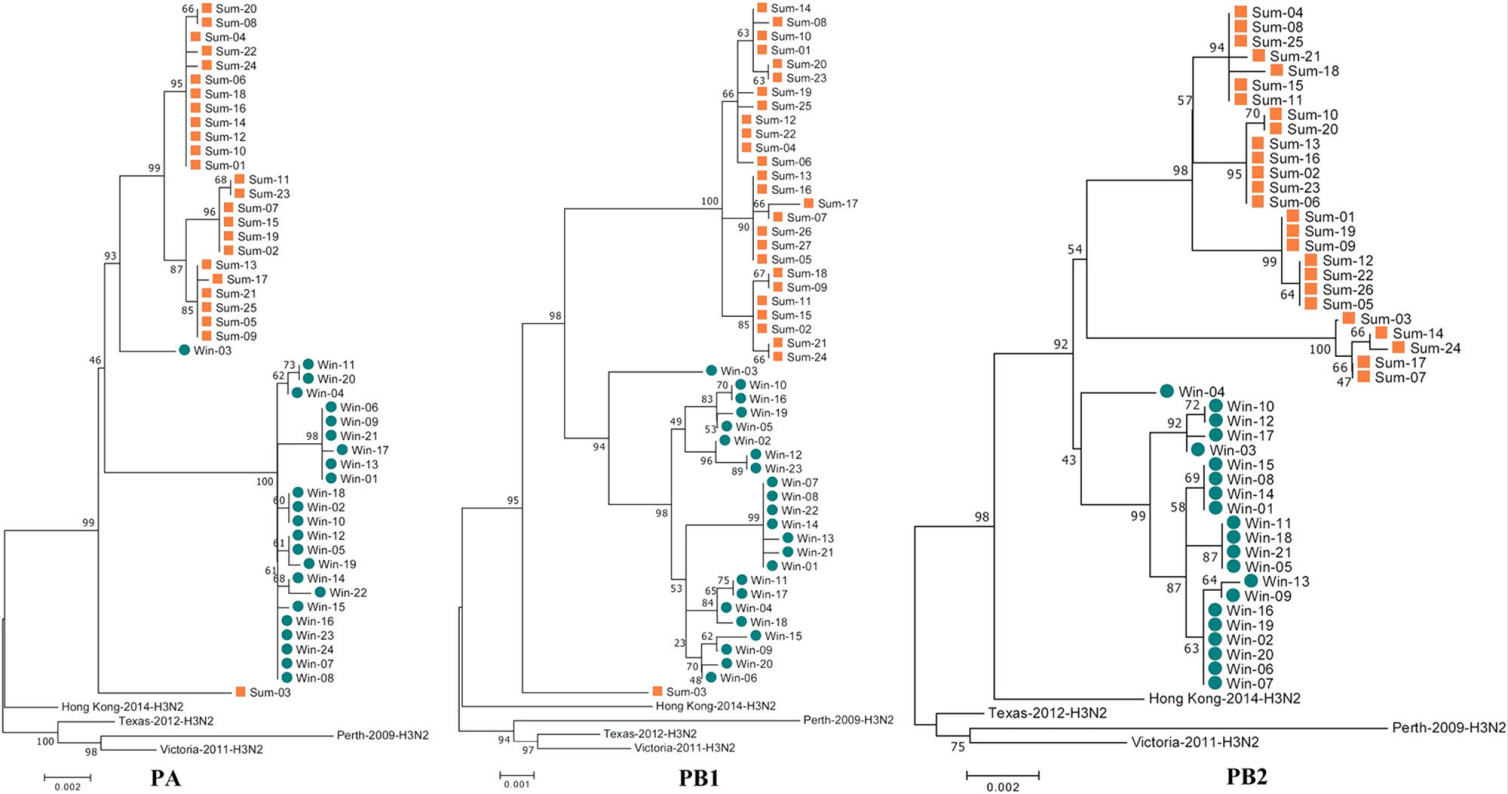
Phylogenetic analysis of the PA, PB1 and PB2 segments circulating between Nov. 2016 and Sep. 2017. The 2016–2017win virus strains are designated Win01–28, and the 2017sum virus strains are designated Sum01–34. The phylogenetic analysis was consistent with the HA and NA analysis

**Fig. 3:**
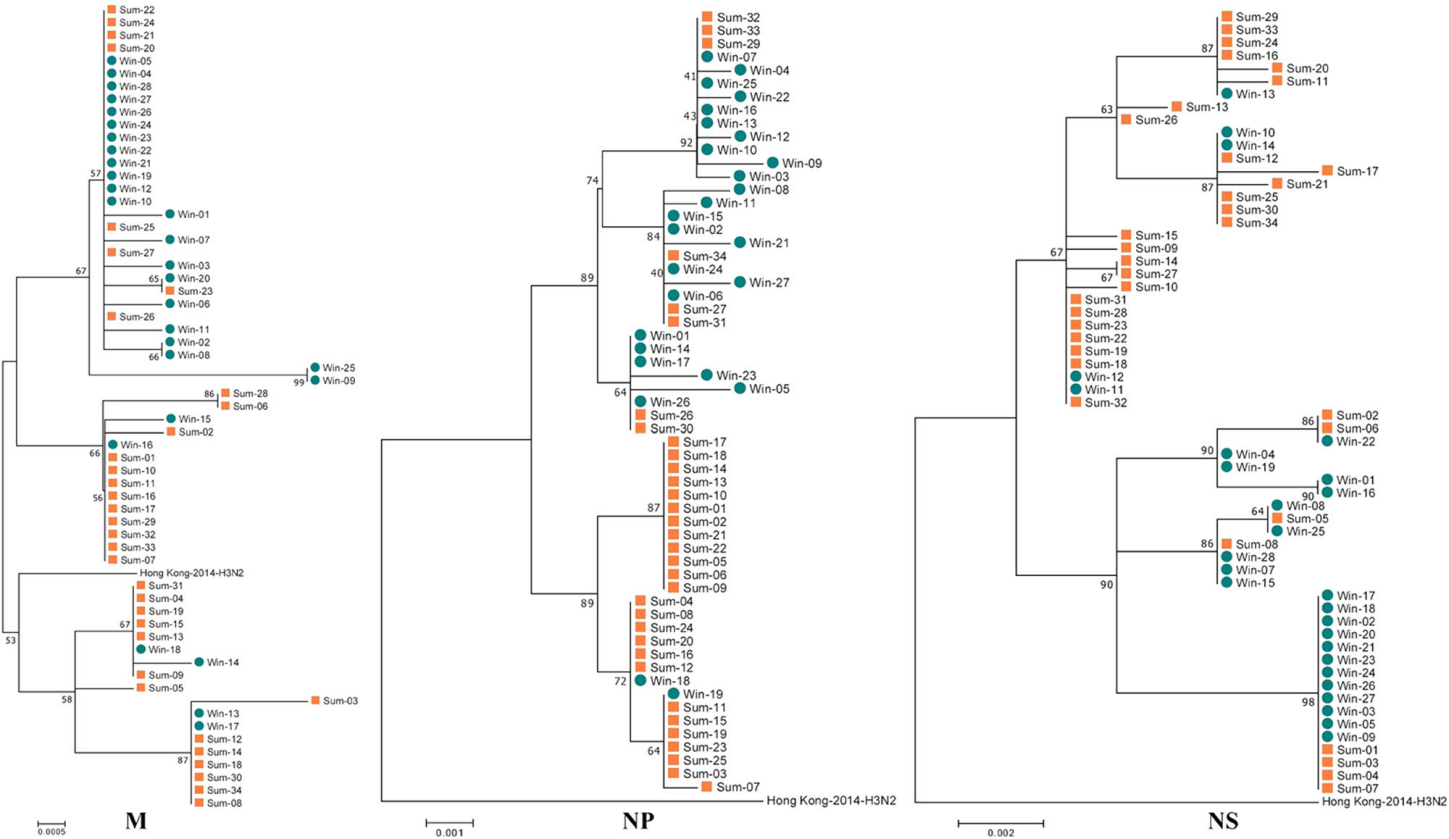
Phylogenetic analysis of the NP, M, and NS segments circulating between Nov. 2016 and Sep. 2017. The 2016–2017win virus strains are designated Win01–28, and the 2017sum virus strains are designated Sum01–34. The phylogenetic analysis was consistent with the analysis of the other segments

The gene sequences of all eight influenza virus gene segments were analyzed. Genetic analysis of the eight segments (PB2, PB1, PA, HA, NP, NA, M, and NS) was performed for all of the samples and for vaccine strains isolated from the Northern Hemisphere between 2010 and 2018. According to the phylogenetic analysis, the 2017sum virus strains significantly differed from the 2016–2017win virus strains, and all of the 2017sum strains evolved from the 2016–2017win strains with several nucleotide alterations (Figs. 1–3). The phylogenetic trees exhibited similar topological structures, with the scale bar ranging from 0.002 to 0.001. The amino acid substitutions in the proteins encoded by these gene segments are summarized in Table 2, including the proportion of each substitution. Most amino acid substitutions were observed in the HA1 subunit. Compared with the WHO-recommended vaccine strains for the 2016–2018 influenza seasons (A/Hong Kong/4801/ 2014), the 2017sum and 2016–2017win virus strains contained 11 amino acid substitutions (T114S, T131K, I140T, R142K, and R261Q in the 2016–2017win strains and K92R, T114S, N121K, T135K/N, N171K, D291E, and H311Q in the 2017sum strains). In addition, the amino acid substitutions between the 2017sum and 2016–2017win strains occurred at positions 220, 303, 329, and 339 in the NA protein; 127 in the NS1 protein; 668 in the PA protein; and 299 and 340 in the PB2 protein.

**Table 2:** Comparison of the 2016–2017win, 2017sum and 2014 H3N2 virus strains with respect to the WHO recommended vaccine strains (A/Hong Kong/4801/2014)

Virus strain	Protein	Amino acid position	Function or domain of mutation (references)	Proportion (%)	Mutations contained in the 2016–2017win isolates (2016–2017win02, 16, 21)	Mutations contained in the 2017sum isolates (2017sum10, 14, 18)	Mutations contained in the 2014 H3N2 virus strain
2016–2017win	PB2	R299K		100	+^a^	–	–
	PB1-F2	R25Q	Pathogenesis associated motif^35^	100	+	–	–
		H75P		100	+	–	–
	PA	I668V	PB1 interaction domain^24^	95.8	+	–	–
	PA-X	R15K		100	+	–	–
	HA1	T114S	HA stalk globular head^36^	100	+	–	+
		T131K		100	+	–	–
		I140T		35.7	–	–	–
		R142K		100	+	–	–
		R261Q		100	+	–	–
	NS1	N127S	PKR-binding domain^26^	100	+	–	–
2017 sum	PB2	K340R		80.8	–	+	–
	PB1-F2	R25Q	Pathogenesis associated motif^35^	100	–	+	–
		H75P		100	–	+	–
	PA-X	R15K		100	–	+	–
	HA1	K92R	HA stalk globular head^31^	91.2	–	+	–
		T114S		91.2	–	+	–
		N121K		100	–	+	–
		T135K		47.1	–	+	–
		T135N		52.9	–	–	–
		N171K		91.2	–	+	
	NA	K220N		96.6	–	+	–
		V303I		100	–	+	–
		N329S		96.6	–	+	–
		D339N		100	–	+	–

^a^Three isolates of each group and wild type virus were chosen as representative strains and the mutations in these strains were listed. Mutation existed: +, mutation didn’t exist: –

For the M2 protein, there was no significant substitution associated with amantadine and rimantadine resistance between the 2017sum and 2016–2017win strains in either the gene or protein sequences. The following amino acid mutations were associated with adamantane resistance: L26F, V27A/T, A30T/V, S31N/R, G34E, A30V, S193F, and R45H^11^. Compared with the vaccine strains from the Northern Hemisphere, no important substitutions were observed in the circulating viral strains during 2016–2017 in the M and NP segments.

### Both the 2016–2017win and 2017sum virus strains replicate efficiently in MDCK cells at 33 °C

**Fig. 4:**
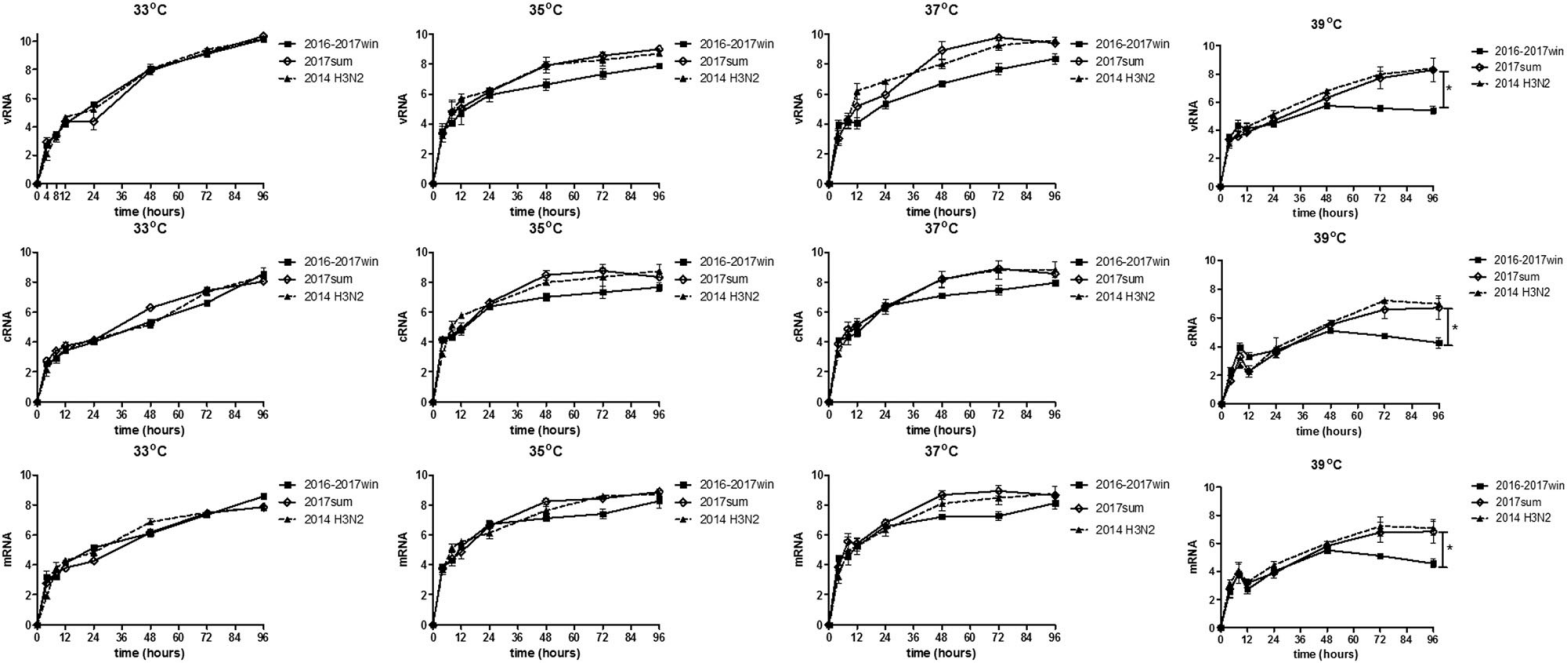
Quantity of vRNA, cRNA and mRNA in infected MDCK cells at 33, 35, 37, and 39 °C. MDCK cells were infected with 2016–2017win, 2017sum and 2014 H3N2 viruses at an MOI of 0.02 and incubated at 33, 35, 37, and 39 °C. The viral vRNA, cRNA, and mRNA of the NP segment in each sample were normalized by a standard curve. The error bars indicate the standard error of the mean. The *P*-values were statistically analyzed by paired *t* test. *P* < 0.05

**Fig. 5:**
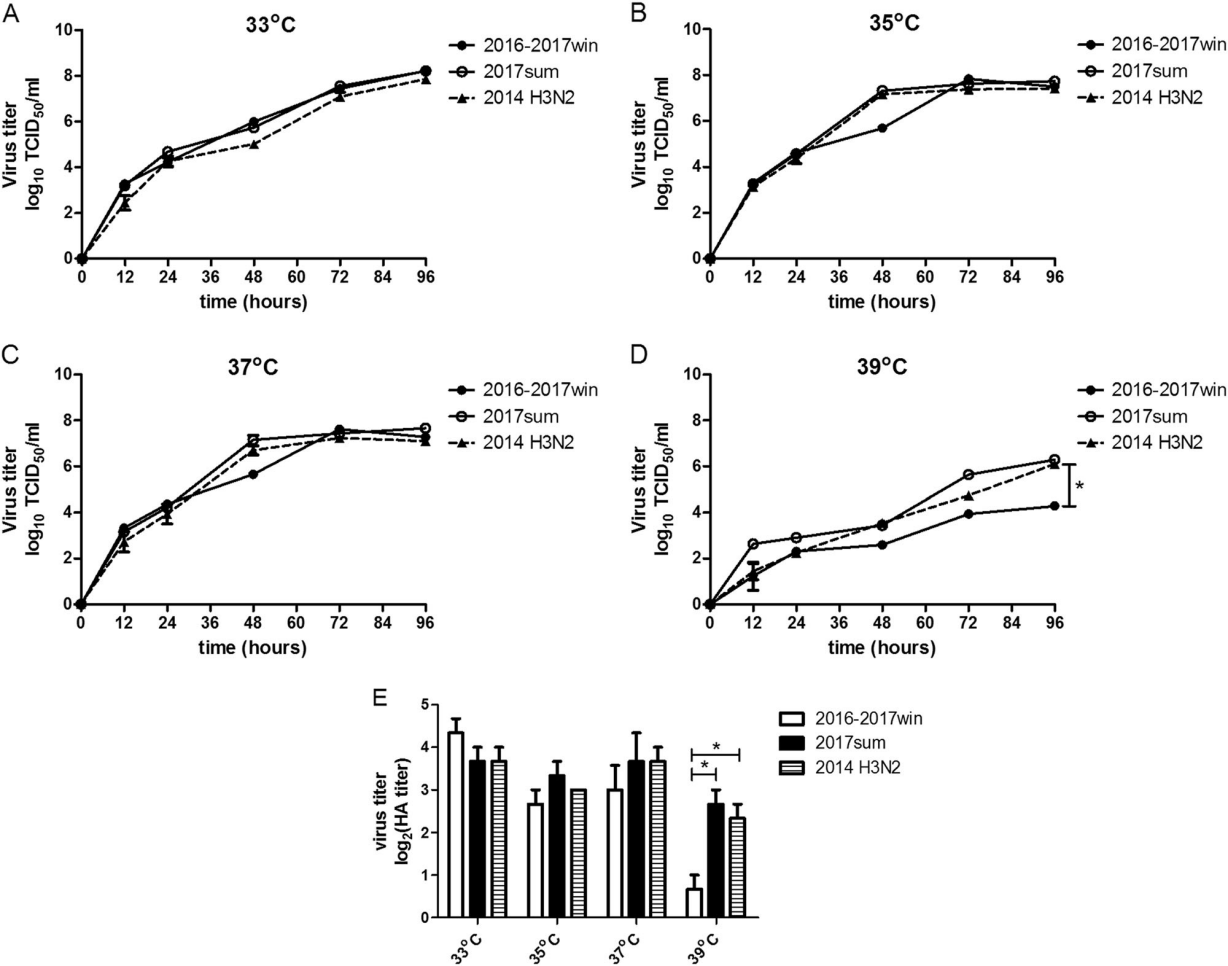
Kinetics of replication by TCID_50_ and the HA assay of 2016–2017win, 2017sum and 2014 H3N2 viruses at 33C, 35, 37, and 39 °C. **a**–**d** The kinetics of replication of the 2016–2017win, 2017sum and 2014 H3N2 viruses at different temperatures were determined according to TCID_50_. **e** Influenza virus replication in MDCK cells at different temperatures was examined by the hemagglutinin assay at 96 hpi. The *P*-values were statistically analyzed by paired *t* test. *P < 0.05

To investigate the mechanism by which seasonal H3N2 mutations influence viral replication, we determined the viral titers based on the TCID_50_ at a series of time points post infection (12, 24, 48, 72, and 96 h) and on the quantities of viral RNA (vRNA), cRNA and viral mRNA in Madin-Darby canine kidney (MDCK) cells infected with the 2016–2017win and 2017sum viral strains at a series of time points post infection (0, 4, 8, 12, 24, 48, 72, and 96 h). Multiple-cycle growth curves of the 2016–2017win, 2017sum and 2014 H3N2 viral strains in MDCK cells are shown in Figs. 4, 5a. All of the viral strains replicated efficiently at 33 °C. There were no significant differences among the replication efficiencies of the 2016–2017win, 2017sum or 2014 H3N2 viruses at 33 °C, with mean titers ranging from 10^7.9^ to 10^8.3^ TCID_50_/ml at 96hpi, and vRNA, cRNA and mRNA were present at high levels in all of the viruses. The HA titers were determined at 96hpi. The results of the HA assay indicated that the 2016–2017win strains may replicate slightly more efficiently than the 2017sum strains at 33 °C (Fig. 5e). The vRNA levels of all of the strains at 96hpi and 33 °C were higher than those at other temperatures (35, 37, and 39 °C).

### Replication of the 2017sum viruses was more efficient than that of the 2016–2017win viruses at 35 °C and 37 °C

The 2017sum virus strains replicated to a mean titer of 10^7.2^ TCID_50_/ml at 35 °C and 10^7.6^ TCID_50_/ml at 37 °C, and the 2016–2017win virus strains replicated to a mean titer of 10^7.5^ TCID_50_/ml at 35 °C and 10^7.3^ TCID_50_/ml at 37 °C (Fig. 5b, c). The final levels of vRNA, cRNA, and mRNA in MDCK cells infected with the 2016–2017win, 2017sum and 2014 H3N2 viruses were relatively similar at 96hpi (Fig. 4). However, the growth rates of the 2016–2017win viruses were lower than those of the 2017sum and 2014 H3N2 viruses, especially after 24hpi. There was an up to 1-log increase in the levels of the 2017sum viruses compared to those of the 2016–2017win viruses at 48 and 72hpi. The RNA levels of the 2017sum and 2014 H3N2 viruses peaked at 72hpi, while the RNA levels of the 2016–2017win viruses peaked at 96hpi. The HA titers of the 2017sum viruses at 35 °C and 37 °C were both higher than those of the 2016–2017win viruses. These data indicated that the 2017sum viruses had higher growth rates and could replicate more efficiently at 35 °C and 37 °C than the 2016–2017win viruses and that replication of the 2016–2017win viruses was attenuated.

### Replication of the 2016–2017win viruses was significantly restricted in MDCK cells at 39 °C

To determine whether the amino acid mutations of the 2016–2017win and 2017sum viral strains influenced the replication of these viruses at high temperatures, we determined the viral titer and RNA levels of the 2016–2017win, 2017sum and 2014 H3N2 viruses at 39 °C. The 2017sum viruses maintained a mean titer of 10^6.3^ TCID_50_/ml at 39 °C, while the 2016–2017win viruses replicated to a mean titer of 10^3.4^TCID_50_/ml at 39 °C (Fig. 5d). The results of the HA assay were consistent with the viral TCID_50_ titers; the HA titer of the 2017sum viruses was significantly higher than that of the 2016–2017win viruses. Meanwhile, the accumulation of vRNA, cRNA, and mRNA in cells infected with the 2017sum viruses was significantly higher than those in cells infected with the 2016–2017win viruses at 39 °C after 24 hpi. RNA replication of the 2016–2017win viruses was restricted by more than 100-fold at 39 °C compared to that at 35 °C and 37 °C. In addition, the peak vRNA levels of the 2016–2017win viruses at 39 °C exhibited a 4-log decrease compared to the RNA levels at 33 °C (Fig. 4). By contrast, the RNA accumulation and growth rates of the 2017sum viruses were stable at different temperatures (33, 35, 37, and 39 °C) and did not exhibit any significant decrease. These data demonstrated that replication of the 2016–2017win viruses was significantly restricted in MDCK cells at 39 °C, indicating that the 2016–2017win virus strains were temperature sensitive.

### Polymerase activity of replication complexes containing PB2 and PA mutations

**Fig. 6:**
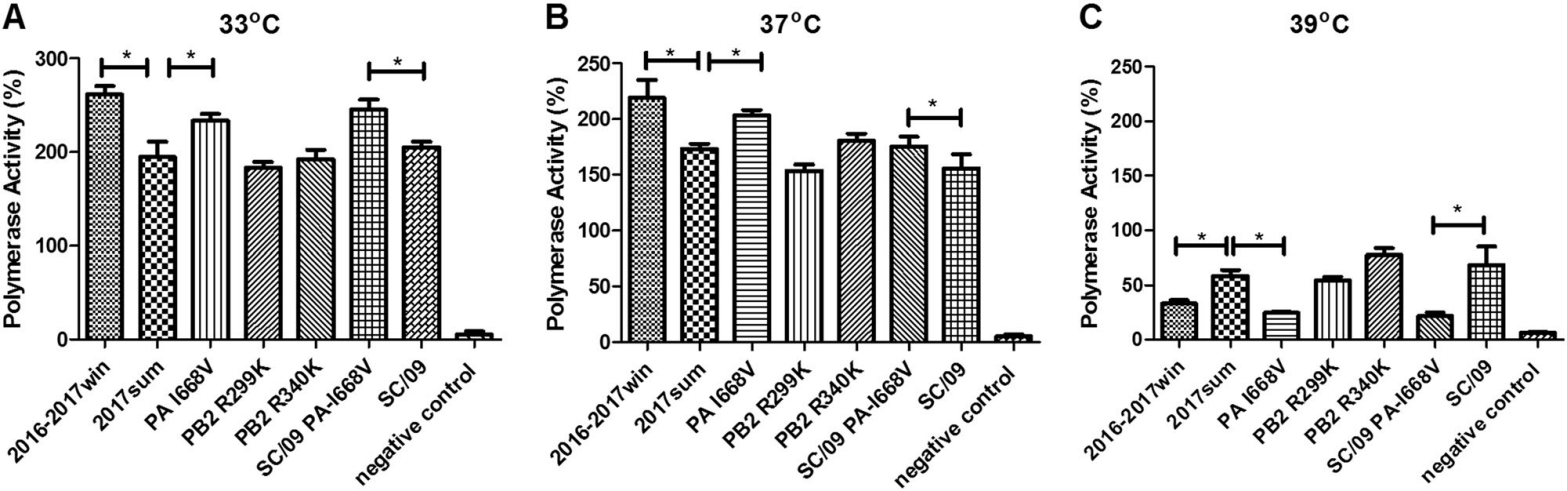
Polymerase activities of the 2016–2017win, 2017sum and SC/09 viruses and their mutations. 293T cells were transfected with a mix of PB2-, PB1-, PA-, and NP-containing plasmids from different virus strains plus a control plasmid and luciferase reporter plasmid. After transfection, 293T cells were cultured at 33 °C (**a**), 37 °C (**b**), and 39 °C (**c**). The polymerase activities were measured in cell lysates 24 h post-transfection. Data are expressed as the mean luciferase activity ± the standard error of the mean of triplicates. The P-values were statistically analyzed by paired *t* test. *P < 0.05

We assessed the effects of the viral polymerase in the 2016–2017win and 2017sum strains on the replication activity using a minireplicon assay. The pPolI-NP-luciferase plasmid produces a modified influenza virus NP viral RNA in which the NP-coding sequences are replaced by the luciferase gene^12,13^. Upon co-transfection of 293T cells with the pPolI-NP-luc plasmid and plasmids expressing PB2, PB1, PA, and NP of the 2016–2017win and 2017sum strains, we observed that the polymerase activity of all of the 2017sum and 2016–2017win strains were higher at 33 °C than those at 37 °C and 39 °C (Fig. 6). The polymerases reconstituted with the PB2, PB1, PA, and NP sequences of the 2016–2017win strain had significantly lower activities than those of the 2017sum strains at 39 °C, whereas the polymerase activities did not differ at 33 °C or 37 °C.

To understand the effects of the PB2 and PA mutations in the 2016–2017win and 2017sum virus strains, we introduced three individual mutations, as summarized in Table 2 (PB2 R299K, PB2 R340K, and PA I668V), into the reassortant polymerase complex from the 2017sum viruses. In human 293T cells, both mutations in PB2 led to similar activities at 33 °C, 37 °C and 39 °C, which was consistent with the polymerase complex of the 2017sum strains. However, the polymerase reconstituted with the PA I668V mutant exhibited significantly reduced activity, a feature that was generally more pronounced at 39 °C than at 33 °C or 37 °C. To verify the decrease in the activity of the PA I668V mutant at 39 °C, an A/Sichuan/1/2009 virus polymerase complex containing the PA I668V mutation (SC/09 PA-I668V) was constructed, and the activity was measured in transfected cells. We compared the activities of the polymerase complexes of the SC/09 virus with the amino acid substitutions in the PA gene via polymerase activity assays. Compared to the polymerase activity of SC/09, the isoleucine-to-valine substitution in the mutant PA I668V of the H1N1 subtype resulted in lower polymerase activity at 39 °C, which was consistent with the PA mutant polymerase complex of the H3N2 subtype (Fig. 6c). These results suggested that the temperature sensitivity of the 2016–2017win viruses was associated with a defect in the replication activity of the polymerase and that the PA I668V mutant might potentially lead to reduced polymerase activity at high temperatures.

## Discussion

Seasonal influenza epidemics are known to result in substantial morbidity and mortality worldwide, particularly in regions with temperate climates, and the mechanism of seasonal influenza activity and transmission has not been elucidated. Influenza seasons are determined by climate regions. Worldwide, influenza epidemics always follow low seasonal temperatures, suggesting that low temperatures may change seasonal crowding, viral survival in respiratory droplets and host susceptibility^14,15^. Moreover, humidity may be a critical driver of influenza outbreaks. Absolute humidity has a regulatory effect on airborne influenza transmission, and this effect can be modulated by temperature^16^. Generally, the northern and southern regions in China experience different influenza seasonality and evolutionary dynamics. Usually, the northern region experiences an influenza season during the winter months (with a median peak in Jan., ranging from Dec. to Jan.), while the southern region experiences influenza during the summer months (with a median peak from May to Jun.). Because Shanghai has a subtropical climate but is located in the intermediate-latitude region, the influenza seasonality in Shanghai is atypical^3,17^. Between 2006 and 2016, the annual seasonality in Shanghai was irregular. Two influenza peaks were observed in 2008 and 2010, and only one epidemic occurred during the other years. However, in 2017, the following two influenza epidemics occurred in Shanghai: one epidemic between Nov. 2016 and Mar. 2017 and one epidemic between May 2017 and Sep. 2017. These epidemics were consistent with the influenza epidemics observed in Hong Kong and Guangdong in 2017. Although influenza epidemics occur frequently during summer in the southern region, the mechanism underlying summer influenza epidemics has not been elucidated^18^. In this study, we performed genomic and replication analyses for viruses obtained during both the winter and summer of 2017 in an attempt to investigate the evolution of the influenza virus during 2017 in Shanghai and the effects of mutations on viral replication in these two epidemic viral strains.

The results of genetic and phylogenetic analyses indicated that the 2017sum virus strains differed from the 2016–2017win virus strains and that there were several amino acid substitutions distributed in different viral proteins. According to the phylogenetic tree of HA, the 2017sum virus strains emerged from the 2016–2017win virus strains. Certain HA segments acted as intermediates during the transition from the cluster of the 2016–2017win viruses to the cluster of the 2017sum viruses, indicating that certain segments of the 2017sum virus strains were already present in influenza viruses circulating in 2016. This tendency was consistent with the phylogenetic analysis of other segments.

The influenza virus polymerase consists of three subunits, encoded by the PA, PB1, and PB2 segments, which also express several important viral proteins, such as PA-X and PB1-F2. Efficient nuclear import and assembly of the polymerase subunit RNP complex are critical steps in the viral life cycle and are closely associated with viral temperature sensitivity^19,20^. In our study, genetic analysis revealed certain unique amino acid substitutions in each group of viral strains. In the PA protein, the 2017sum strains exhibited a V668I substitution located in the PB1 interaction domain. The 2017sum viral strains differed from the 2016–2017win viral strains in regard to the K299R and K340R mutations in the PB2 protein. To further investigate the effects of these mutations on the influenza polymerase in the 2016–2017win and 2017sum virus strains, we evaluated viral replication via a TCID_50_ measurement, an HA assay and the vRNA, cRNA, and mRNA levels of the NP segment in MDCK cells infected with these two viral strains at different temperatures (33, 35, 37, and 39 °C). Our data showed that replication of the 2016–2017win viruses was significantly restricted at 39 °C. A previous study demonstrated the role of the polymerase complex in determining the temperature range over which influenza viruses can replicate^5^. Bradel Tretheway et al.^21^ showed that the human WSN polymerase complex lost activity at 39 °C. The shutoff temperature of most temperature-sensitive viruses is 39 °C or higher. These conclusions are consistent with our results for the 2016–2017win strains. In this study, replication of the 2016–2017win strains was similar to that of temperature-sensitive viruses, exhibiting high viral replication at 33 °C and significantly low replication at 39 °C.

Furthermore, we demonstrated that the temperature sensitivity of the 2016–2017win viruses was conferred by a mutation in the influenza polymerase PA subunit. Introducing a single substitution at residue 668 of the PA gene induced a strong attenuation of polymerase activity at 39 °C, whereas the substitutions at residues 299 and 340 of PB2 had little influence on the polymerase activity at different temperatures. The mechanism by which the PA I668V mutation suppressed polymerase activity at high temperatures was not elucidated. Temperature-sensitive mutations fall into two general classes: those generating thermolabile proteins and those generating defects in protein synthesis, folding or assembly^22^. The PA amino acid 668 maps to a helix located in the C-terminal region of the PA protein^23^, suggesting that this mutation may belong to the latter class. Previous research has confirmed that this helix (residues 657–716) is required for binding to the N terminus of PB1, suggesting that residue 668 may be involved in protein–protein interactions and may affect polymerase assembly^24,25^. This finding is consistent with our results showing that polymerases of both the H1N1 and H3N2 viruses with the PA I668V residue exhibit reduced activity at high temperatures. The I668V mutation may regulate the PA-PB1 interaction or inhibit polymerase function.

In addition, there was a decrease in the accumulation rate of vRNA, cRNA, and mRNA in MDCK cells infected with the 2016–2017win viruses at 35 °C and 37 °C compared with that in cells infected with the 2017sum and 2014 H3N2 viruses after 24 hpi; this result was consistent with those from the TCID_50_ measurement and HA assay. Combined with our genome-wide analysis, we found one mutation in the NS gene that might result in a decrease in the growth rate of the 2016–2017win viruses. The N127S mutation identified in NS1 has not been reported previously; however, this position had been confirmed to be part of the PKR-binding site of the NS1 protein (position 123–127)^26^. The influenza virus NS1 protein plays a key role in blocking the activation of PKR during influenza infection and interacts with the viral polymerase to mediate the temporal regulation of vRNA synthesis during infection. Mutants of NS1 with mutations at position 123/124 or 126/127 do not bind PKR, and mutations at position 123/124 can result in the deregulation of vRNA synthesis, resulting in attenuated influenza virus replication^27^. There has been no report regarding mutations at position 127 of NS1, but our data were consistent with the results of the analysis of mutations at position 123/124 of the NS1 protein. We hypothesize that the decrease in the RNA accumulation and TCID_50_ value in cells infected with the 2016–2017win viruses may result from the N127S mutation in the NS1 protein. Viruses with the N127S mutation may lose the ability to interact with the viral polymerase and exhibit low synthesis of vRNA at the early infection stage.

In summary, the influenza A (H3N2) virus strains circulating in the 2016–2017 winter season and 2017 summer season in Shanghai were clustered into two different groups according to a phylogenetic analysis. The 2016–2017win virus strains were temperature sensitive and attenuated compared with the 2017sum virus strains, and the 2017sum viruses exhibited an adaptation to high temperatures. A mutation in PA (at position 668) appeared to be critical for replication and temperature sensitivity. Adaptation to high temperatures may be the result of the natural evolution of the influenza virus and could explain the second epidemic that occurred during the summer of 2017 in Shanghai. These data further enhance our understanding of the summer influenza epidemic in Shanghai and subtropical Asia and thus provide insights for the prediction of the next epidemic strain of influenza A (H3N2). Our research provides information on the temperature-sensitive signature and further insight into the function of the influenza A polymerase. Future studies investigating the functional properties of viral polymerases should be performed to fully understand the evolution of the influenza virus and pave the way for the prevention of new severe influenza pandemics.

## Materials and methods

### Ethics statement

The Research Laboratory of Clinical Virology of Ruijin Hospital collected human nasopharyngeal swab samples from residual samples that had been obtained for clinical respiratory virus detection. Each patient received a clinical report and was informed of the result of the laboratory diagnostic test before our study began. All procedures complied with the Measures for the Ethical Review of Biomedical Research Involving Human Subjects issued by the National Health and Family Planning Commission of The People’s Republic of China. The Ruijin Hospital Ethics Committee, Shanghai Jiaotong University School of Medicine, approved the study protocol (permit number: Ruijin Hospital Ethics Committee 2018–48). All experimental infection procedures were performed in biosafety level 2 facilities.

### Sample collection and virus isolation

All samples were obtained from the Research Laboratory of Clinical Virology, Institute of Infectious and Respiratory Diseases, Ruijin Hospital. Nasopharyngeal specimens from the 2016–2017 winter and 2017 summer influenza outbreaks were collected from influenza-infected inpatients before the administration of antiviral treatment. The samples were classified into the following two groups in chronological order: 2016win (Nov. 2016 to Mar. 2017) and 2017sum (May 2017 to Sep. 2017). Nasopharyngeal swabs were placed into 2 ml cryovials containing virus transport medium and stored at −80 °C until isolation and analysis.

All samples were identified and subtyped by performing real-time RT-PCR. Eight complete gene segments of 47 samples from 62 influenza-infected inpatients were amplified successfully. For the other 15 samples, we analyzed only partial viral genomes because of the low levels of vRNA. In total, 448 gene segments were included in our genome-wide analysis, including the 47 PB2, 50 PB1, 49 PA, 62 HA, 59 NP, 57 NA, 62 M, and 62 NS segments. Among these 62 samples, 28 were obtained from inpatients between Nov. 2016 and Mar. 2017 (2016–2017win 1–28) and 34 were obtained from inpatients between May 2017 and Sep. 2017 (2017sum 1–34). All segments were submitted to the Influenza Research Database under GenBank accession numbers MG984093 to MG984541.

The influenza A viruses were isolated from MDCK cells as previously described^28^. We chose six isolates in this study for viral growth and replication kinetic assays: three isolates represented the 2016–2017win virus strains (2016–2017win02, 16, 21) and three isolates represented the 2017sum virus strains (2017sum10, 14, 18). The isolates from each group shared more than 99.9% identity in all eight segments. We used an influenza A (H3N2) viral strain isolated in 2014 as our control virus. This control virus strain, isolated in Ruijin Hospital in Dec. 2014, was highly homologous (>99%) to the WHO-recommended vaccine strains A/Texas/50/2012 (H3N2) and was designated as 2014 H3N2. The whole-genome sequences of the 2014 H3N2 viral strains was determined and submitted to GISAID (accession number EPI1230942-EPI1230949).

### Plasmids

A/Sichuan/01/2009 (H1N1) virus (SC/09) cDNA in pBD vectors was kindly provided by Hualan Chen (Harbin Veterinary Research Institute, Chinese Academy of Agricultural Sciences, China), and the luciferase reporter plasmid pPolI-NP-luc was provided by Ke Xu (Institute Pasteur of Shanghai, Shanghai, China). The H3N2 polymerases of the 2016–2017win and 2017sum strains were generated using plasmid-based reverse genetic techniques. The PB2, PB1, PA, and NS viral segments from these two viral strains were cloned into bidirectional pBD vectors. Mutations in PB2 (R229K, R340K) and PA (I668V) were introduced by site-directed mutagenesis.

### vRNA amplification and sequencing

vRNA was extracted using an AxyPrep Body Fluid Viral DNA/RNA Miniprep Kit (Corning Life Sciences, New York, USA) according to the manufacturer’s instructions. Contaminant DNA was eliminated using DNase. Amplification of all eight influenza genome segments (PB2, PB1, PA, HA, NP, NA, M, and NS) was performed using a HiScript II One Step RT-PCR Kit (Vazyme Biotech, Nanjing, China) and a set of primers (Table S1). Amplification was performed under the following conditions: initial reverse transcription at 50 °C for 15 min, followed by 40 cycles of denaturation at 94 °C for 5 min, denaturation at 94 °C for 15 s, annealing at 50 °C for 30 s and extension at 72 °C for 2 min. All of the PCR products were purified using a QIAquick Gel Extraction Kit (Qiagen, Hilden, Germany). DNA sequencing was performed using the dideoxy chain termination method using a TaqDyeDeoxy Terminator Cycle-Sequencing Kit (Applied Biosystems, Foster City, USA) according to the manufacturer’s instructions. The sequencing reactions were analyzed on an ABI 3730XL DNA analyzer (Applied Biosystems, Foster City, USA).

### Phylogenetic analysis

The sequences of the reference strains and Northern Hemisphere vaccine strains recommended by the WHO that were included in the phylogenetic analysis were downloaded from the GISAID database. MEGA version 7.0 was used to construct phylogenetic trees using the maximum likelihood method under the general time reversible + proportion of invariant sites + shape parameter of the γ distribution (GTR + I + Γ) model as described^29,30^. The amino acids in epitopes A to E of the influenza A (H3N2) HA protein referenced in this study have been previously identified^30^.

### Viral growth and replication kinetics

MDCK cells were infected with the 2014 H3N2, 2016–2017win, and 2017sum viruses at a multiplicity of infection (MOI) of 0.02 at 33 , 35, 37 or 39 °C. After 1 h of virus adsorption at room temperature, the cells were washed twice with PBS and overlaid with DMEM containing 0.3% BSA, 100 IU/ml penicillin and 2 μg/ml TPCK-treated trypsin^31^. At the indicated times (0, 4, 8, 12, 24, 48, 72, and 96hpi), the culture supernatants were collected, and the viral titers and replication kinetics were determined by a TCID_50_ measurement (0, 12, 24, 48, 72, and 96hpi) and real-time PCR assay (0, 4, 8, 12, 24, 48, 72, and 96hpi). The dilution at which 50% of the wells were infected (TCID_50_) was determined as described previously^32^, and the titers were expressed as log_10_ TCID_50_/ml.

### Quantification of vRNA, cRNA, and mRNA in infected MDCK cells

Total RNA was extracted and reverse transcribed according to the Hot-Start protocol as previously described^33^. Unique tags consisting of an additional 18 to 20 nucleotides unrelated to the influenza virus were added to distinguish the three types of influenza RNAs (vRNA, cRNA, and mRNA). The resulting cDNA was amplified by quantitative PCR with an ABI Prism 7500 system (Applied Biosystems, Life Technologies) using SYBR Green SuperMix (Vazyme Biotech, Nanjing, China). The primers used are listed in Supplementary Table S2. Ten-fold serial dilutions (10^8^, 10^7^, 10^6^, 10^5^, 10^4^, 10^3^ copies/μl) of the vRNA plasmids were used to generate a standard curve.

### HA assay

The HA assay was performed as previously described^34^. Briefly, the cell culture supernatants of infected cells at 96hpi were serially diluted with PBS, and an equal volume of 1% cavy erythrocytes was added. The viral titers were calculated 40 min later.

### Luciferase assay of viral polymerase activity

Monolayers of 293T cells were transfected with 125 ng of the luciferase reporter plasmid pPolI-NP-luc and 12.5 ng of the internal control plasmid pRL-CMV (Promega, Wisconsin, USA) with a mix of PB2-, PB1-, PA-, and NP-containing plasmids at quantities of 125, 125, 125, and 250 ng, respectively. The transfected 293T cells were incubated at 33, 37, and 39 °C and lysed 24 h post transfection. The luciferase activity was analyzed with a dual-luciferase assay system (Promega, Wisconsin, USA) according to the manufacturer’s instructions. The results were the averages from three independent experiments.

### Statistical analysis

Qualitative and quantitative variables were compared between the two groups (2016–2017win and 2017sum) using the Chi-squared test and one-way analysis of variance (one-way ANOVA). No adjustment for multiple testing was performed. The levels of the replication and polymerase activity were statistically analyzed by a paired *t* test. Statistical analysis was performed using SPSS 18.0 (SPSS, Inc., Chicago, USA).

## Electronic supplementary material

Table S1

Table S2
