# Supplementary material for: Genome-wide characterization of the seasonal H3N2 virus in Shanghai reveals natural temperature-sensitive strains conferred by the I668V mutation in the PA subunit
Source: Emerg Microbes Infect. 2018 Oct 23;7:171. doi: 10.1038/s41426-018-0172-4 (PMC6199244; doi:10.1038/s41426-018-0172-4)
Supplement: Supplementary file 1 — Table S1 [file 41426_2018_172_MOESM1_ESM.docx]

**Table S1 Sequences of primers for influenza A(H3N2) virus in Shanghai from 2016-2017**

| Segment | Primer | Sequence 5’-3’ |
| --- | --- | --- |
| PB2 | PB2-F | TCAATTATATTCAGCATGGA |
|  | PB2-R | TCGTTTTTAAACTATTCAGT |
| PB1 | PB1-F | CAAACCATTTGAATGGATGT |
|  | PB1-R | CATTTTTTCATGAAGGACAA |
| PA | PA-F | TACTGATTCAAAATGGAAGA |
|  | PA-R | TACTTTTTTGGACAGTACGG |
| HA | HA-F | GGATAATTCTATTAACCATGA |
|  | HA-R | GTGTTTTTAATTAATGCACT |
| NP | NP-F | GTTGATAATCACTCACTGAGT |
|  | NP-R | GAAGAAATAAGATCCTTCGT |
| NA | NA-F | AGTAAAGATGAATCCAAATCA |
|  | NA-R | AGTTTTTTCTAAAATTGCGA |
| M | M-F | ATGAGCCTTCTAACCGAGGTC |
|  | M-R | TCCAACTCTATGCTGACAAAA |
| NS | NS-F | GTGACAAAGACATAATGGATTC |
|  | NS-R | GTAGAAACAAGGGTGTTTTTTA |
