# Supplementary material for: Genome-wide characterization of the seasonal H3N2 virus in Shanghai reveals natural temperature-sensitive strains conferred by the I668V mutation in the PA subunit
Source: Emerg Microbes Infect. 2018 Oct 23;7:171. doi: 10.1038/s41426-018-0172-4 (PMC6199244; doi:10.1038/s41426-018-0172-4)
Supplement: Supplementary file 2 — Table S2 [file 41426_2018_172_MOESM2_ESM.docx]

**Table S2 Sequences of primers for quantification of viral RNA, cRNA and mRNA in infected MDCK cells.**

| Primer | Segment | Sequence 5’-3’ |
| --- | --- | --- |
| H3-NP vRNA re | NP | GGCCGTCATGGTGGCGAAT GAATGGGCGGAAAACAAGAAGTGC |
| H3-NP vRNA tag | NP | GGCCGTCATGGTGGCGAAT |
| H3-NP vRNA F | NP | CTCAATATCAGTGCAGATCTTGCT |
| H3-NP cRNA re | NP | GCTAGCTTCAGCTAGGCATC GTATTTTTTCCTTAATTGTCGTACTCTT |
| H3-NP cRNA tag | NP | GCTAGCTTCAGCTAGGCATC |
| H3-NP cRNA f | NP | CGATCGTGCCCTCTTTTG |
| H3-NP mRNA re | NP | CCAGATCGTTCGAGTCGT TTTTTTTTTTTTTTTTCCTTAATTGTC |
| H3-NP mRNA tag | NP | CCAGATCGTTCGAGTCGT |
| H3-NP mRNA f | NP | CGATCGTGCCCTCTTTTG |
